# Dolutegravir–lamivudine as initial therapy in HIV-1 infected, ARV-naive patients, 48-week results of the PADDLE (Pilot Antiretroviral Design with Dolutegravir LamivudinE) study

**DOI:** 10.7448/IAS.20.01.21678

**Published:** 2017-05-10

**Authors:** Pedro Cahn, María José Rolón, María Inés Figueroa, Ana Gun, Patricia Patterson, Omar Sued

**Affiliations:** ^a^ Clinical Research Department, Fundación Huésped, Buenos Aires, Argentina; ^b^ Laboratory, Clinical Research Department, Fundación Huésped, Buenos Aires, Argentina

**Keywords:** dual therapy, dolutegravir, lamivudine, naive patients

## Abstract

**Introduction**: A proof-of-concept study was designed to evaluate the antiviral efficacy, safety and tolerability of a two-drug regimen with dolutegravir 50 mg once daily (QD) plus lamivudine 300 mg once daily as initial highly active antiretroviral therapy (HAART) among antiretroviral (ARV)-naive patients.

**Methods**: PADDLE is a pilot study including 20 treatment-naive adults. To be selected, participants had no IAS-USA-defined resistance, HIV-1 RNA ≤100,000 copies/mL at screening and negative HBsAg. Plasma viral load (pVL) was measured at baseline; days 2, 4, 7, 10, 14, 21 and 28; weeks 6, 8 and 12; and thereafter every 12 weeks up to 96 weeks. Primary endpoint was the proportion of patients with HIV-1 RNA <50 copies/mL in an intention to treat (ITT)-exposed analysis at 48 weeks (the FDA snapshot algorithm).

**Results**: Median HIV-1 RNA at entry was 24,128 copies/mL (interquartile range (IQR): 11,686–36,794). Albeit as per protocol, all patients had pVL ≤100,000 copies/mL at screening as required by inclusion criteria, four patients had ≥100,000 copies/mL at baseline. Median baseline CD4+ T-cell count was 507 per cubic millimetre (IQR: 296–517). A rapid decline in pVL was observed (median VL decay from baseline to week 12 was 2.74 logs). All patients were suppressed at week 8 onwards up to week 24. At week 48, 90% (18/20) reached the primary endpoint of a pVL <50 copies/mL. Median change in CD4 cell count between baseline and week 48 was 267 cells/mm^3^ (IQR: 180–462). No major tolerability/toxicity issues were observed. Nineteen patients completed 48 weeks of the study, and one patient (with undetectable VL at last visit) committed suicide. One patient presented a low-level protocol-defined confirmed virological failure at week 36, being the only observed failure. This patient had pVL <50 copies/mL at the end-of-study visit without having changed the two-drug regimen. Observed failure rate was 5%. This is the first report of integrase strand transfer inhibitor/lamivudine dual regimen in ARV-naive patients.

**Conclusions**: This novel dual regimen of dolutegravir and lamivudine warrants further clinical research and consideration as a potential therapeutic option for ARV-therapy-naive patients.

**ClinicalTrials.gov Identifier**: NCT02211482.

## Introduction

Zidovudine (ZDV) monotherapy was the first attempt to control HIV replication. Soon after, two-drug combination became the preferred strategy, as ZDV effects lasted only for the short term, due to selection of resistance mutations. Unfortunately, the dual strategy also failed in achieving long-lasting virologic control. Combination antiretroviral therapy (ART) containing three active drugs from at least two different classes has been the standard of care for HIV treatment all over the world since 1996, based on the findings of two seminal studies [1,2].

Expansion of access to antiretroviral (ARV) therapy has been the main driver of a striking 38% decline in HIV incidence and a 35% reduction in AIDS-associated mortality since 2000 [3]. Current HIV treatment guidelines recommend first-line ARV regimens consisting of two nucleoside/nucleotide analogue reverse-transcriptase inhibitors (NRTIs) as a “backbone” combined with a third agent from the non-nucleoside reverse-transcriptase inhibitor, the boosted protease inhibitor (PI), or the integrase strand transfer inhibitor (INSTI) classes. Over the past two decades, novel drugs for ART have improved the rates of long-term viral suppression, but even the most widely used regimens might result in treatment modification or interruption because of tolerability, toxicities or issues like challenging treatment schedules, drug interactions and food requirements [4].

To reduce toxicity, complexity, and costs, strategies that decrease the number of active drugs have been evaluated, both as initial therapy and as maintenance therapy for patients who achieved virologic suppression. Most efforts have yielded unacceptable high rates of treatment failure [5]. A two-drug regimen with a boosted PI plus lamivudine (3TC) has demonstrated favourable results in treatment-naive patients. The GARDEL study was the first to show non-inferiority of a dual combination of ritonavir-boosted lopinavir (LPV/r) plus 3TC, even in patients with baseline viral load (VL) above 100,000 copies/mL [6]. Other three studies showed non-inferior virologic efficacy of the same dual regimen or the combination of ritonavir-boosted atazanavir plus 3TC, all performed in virologically suppressed patients as a switch strategy [7–9].

Dolutegravir (DTG) is a potent INSTI, exhibiting rapid reduction in VL and a high barrier to resistance. DTG is a QD drug, well tolerated, that can be taken with or without food [10], with a low potential for drug–drug interactions [11], and a high genetic barrier [12]. Comparison with other preferred first-line drugs showed non-inferiority of DTG to raltegravir and superiority to efavirenz (EFV) and darunavir (DRV) in the randomized clinical trials SPRING, SINGLE and FLAMINGO, respectively [13–15].

Lamivudine (3TC) is a potent cytidine nucleoside analogue without major side effects, and it has a well-proven safety profile. In monotherapy, it has shown VL reduction up to 1.19 log [16]. It has a low genetic barrier selecting, in case of failure, the M184V mutation, which has been linked to a reduction in viral fitness [17]. This NRTI is also a QD drug, it can also be administered with or without food and no clinically relevant drug–drug interactions have been reported with its use [18].

The purpose of this pilot study was to assess the antiviral efficacy, safety and tolerability of a two-drug regimen with DTG 50 mg QD plus 3TC 300 mg QD as initial HAART among ARV-naive patients.

## Methods

PADDLE is an ongoing pilot study involving HIV-1-infected patients naive to ARV treatment. Participants were enrolled in Argentina between 24 September 2014 and 28 February 2015. The last participant completed 48 weeks of treatment on 24 February 2016.

This study was conducted in accordance with good clinical practice procedures, all applicable regulatory requirements and the guiding principles of the Declaration of Helsinki. The study protocol was reviewed and approved by the Institutional Review Board (Comité de Bioética, Fundación Huésped). All patients provided written informed consent before entering the study.

## Study design and participants

Eligible participants were 18 years of age or older, had HIV-1 infection, were naive to ARV treatment (never exposed to ARV drugs), had a plasma HIV-1 RNA level at screening visit >5000 copies and ≤100,000 copies per millilitre and a CD4+ T-cell count >200 per cubic millimetre with no evidence of genotypic of viral resistance to lamivudine as per IAS-USA 2013 resistance panel [19].

Main exclusion criteria were previous exposure to ARVs, VL >100,000 copies/mL, CD4 count below 200 cells/mL at screening, pregnancy or breastfeeding, severe hepatic impairment, grade 4 laboratory abnormalities or alanine aminotransferase (ALT) >5 times the upper limit of normal (ULN) or ALT ≥3 times the ULN and bilirubin ≥1.5 times the ULN (with >35% direct bilirubin) or creatinine clearance of <50 mL/min via Cockroft–Gault method.

All participants were assigned to receive DTG 50 mg once daily plus 3TC 300 mg once daily.

In order to ensure patient’s safety, a phase 2 implementation protocol was designed: In phase 1, 10 patients were included. If, at week 8, at least 8 out of 10 patients showed a VL decrease >1 log, the study would continue to phase 2 in which another 10 patients would be enrolled. If more than two patients showed a VL decrease <1 log, the study would be discontinued. In addition, all patients would have an intensive follow-up. Patients would continue on study unless any of the following stopping rules were met: Patients with VL ≥1000 copies/mL at week 12, patients with VL ≥400 copies/mL at week 24 or 36 or patients with a confirmed viral rebound (>200 c/mL) after VL <50 c/mL.

## Procedures

Study visits were scheduled at baseline; days 2, 4, 7 and 10; and weeks 2, 3, 4, 6, 8, 12, 24, 36 and 48. On completion of the week 48 visits, all the participants were offered the opportunity to continue in a study extension until week 96. The Abbott Real-Time HIV-1 assay was used to detect the HIV-1 RNA plasma level (lower limit of detection, 40 copies per millilitre). Adverse events (AEs), serious adverse events (SAEs) and laboratory measurements (including haematological measurements, fasting lipid profile and blood-chemistry profile) were assessed intermittently throughout the study, and their severity graded according to the criteria of the Division of AIDS (DAIDS) at the National Institute of Allergy and Infectious Diseases of the National Institutes of Health. Specifically, safety evaluations were completed as follows: AEs and concomitant medications on all visits and laboratory parameters and vital signs on screening, baseline and weeks 2, 4, 8, 12, 24, 36 and 48. Viral genotype was analysed (TRUGENE HIV-1 Genotyping Kit) at the screening visit and had to be obtained at time of protocol-defined virologic failure (PDVF) defined as VL ≥1000 copies/mL at week 12, VL ≥400 copies/mL at week 24 or 36 or a confirmed viral rebound (>200 c/mL) after VL <50 c/mL.

Participants were required to withdraw from the study if PDVF was confirmed. Baseline was assessed with the AIDS Clinical Trials Group (ACTG) Adherence/Baseline questionnaire at baseline visit and with ACTG/Follow-up Questionnaire at visits: 4, 8, 12, 24, 36 and 48.

## Outcomes

The primary efficacy endpoint was the proportion of subjects with plasma HIV-1 RNA below 50 copies per millilitre at week 48 using the FDA snapshot algorithm (Missing, Switch or Discontinuation = failure) for the intention-to-treat exposed population (described in the Statistical analysis section). Secondary safety and efficacy endpoints included the frequency, type and severity of AEs and laboratory abnormalities, the proportion of patients with HIV-1 RNA <1000 copies/mL at week 12, the proportion of patients with HIV-RNA <400 at week 24, the incidence of genotypic and phenotypic resistance in case of virologic failure, the change from baseline in the CD4+-cell count, the lipid profile changes between baseline and week 48 and the viral decay rate.

## Statistical analysis

Virological efficacy is shown as intention-to-treat (exposed) and as per protocol. Final analysis includes 48-week results for the 20 patients. Descriptive statistics were used to evaluate the primary and secondary outcomes. The results are given as median and interquartile ranges (IQRs) or frequencies (%) as appropriate. Changes in CD4 were analysed similarly. All AEs were evaluated, summarized and described as the proportion of patients presenting events and the total number of events. In all samples with plasma HIV-RNA below limit of detection, absence of signal was tested.

## Role of the funding source

The study was sponsored by Fundación Huésped. Trial funding was provided by ViiV Healthcare. ViiV Healthcare contributed towards scientific review of the study design and this report. All operational aspects of the study, including study design, monitoring, data collection, data analysis and writing of the report, were managed by Fundación Huésped. All the authors had full access to all the data in the study and are responsible for the veracity and completeness of the data reported. The corresponding author has final responsibility for the decision to submit for publication.

## Results

**Figure 1.**
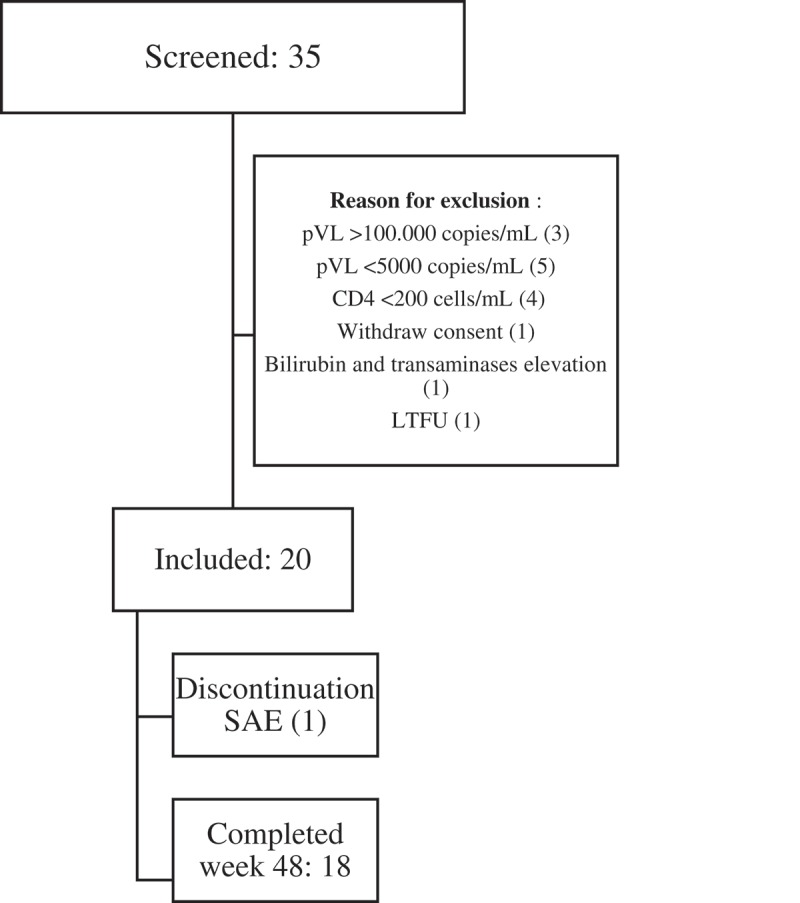
Trial profile.

A total of 35 patients were screened, and 20 patients were included and treated (Figure 1).

Demographic and baseline disease characteristics are referred to in Table 1. The median HIV-1 RNA level at baseline was 24,128 copies/mL (IQR: 11,686–36,794). Albeit, as per protocol, all patients had pVL <100,000 copies/mL, four patients had ≥100,000 copies/mL at baseline and entered the study (Table 1).

**Table 1.** Demographic and baseline disease characteristics.

Demographic and baseline characteristics	
Gender (male: female)	19:1
Age, years, median (IQR)	34 (31–43)
Mode of transmission (*n*) MSM Heterosexual	15 5
HIV-RNA (copies/mL), median (IQR)	24,128 (11,686–36,794)
CD4 count, cells/mm^3^, median (IQR)	507 (296–517)
CDC stage (%) A/B/C	90/10/0

IQR: interquartile range.

CDC: Centers for Disease Control and Prevention.

All but one patient, who committed suicide, completed 48 weeks in the study and were evaluated for the primary endpoint. Figure 1 shows the disposition of patients during the trial. Adherence (self-reported and controlled by pill counts at each visit) was 100%. Median CD4 cell count change from baseline was 267 cells/mm^3^ (IQR: 180–462).

**Figure 2.**
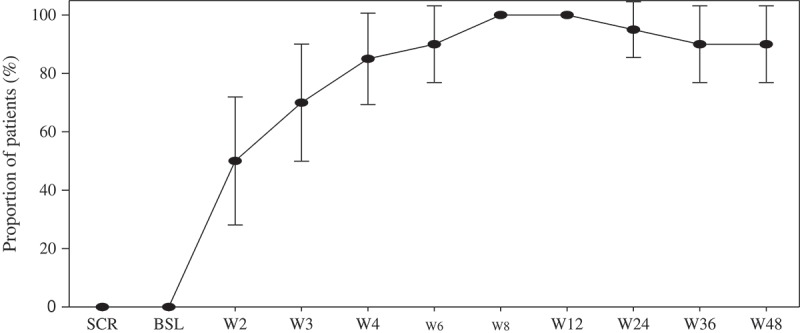
Proportion of patients with plasma HIV-1 RNA lower than 50 copies per mL, by visit (Snapshot analysis). Analysis included all participants who received at least one dose of study drug.

At week 48, 18 out of 20 subjects (90%, 95% confidence interval (CI): 77–100), reached plasma HIV-1 RNA level of less than 50 copies per millilitre in ITT-exposed analyses (Figure 2).

In the per-protocol analyses, 18 out of 19 subjects were responders (95%, 95% CI: 85–100). All subjects, including the four subjects that entered the study with baseline pVL above 100,000 copies/mL, achieved VL below 400 copies at week 3 and HIV-1 RNA level of <50 copies per millilitre at 8 weeks.

A rapid antiviral response was observed (median VL decay from baseline to week 12 was 2.74 logs).

**Figure 3.**
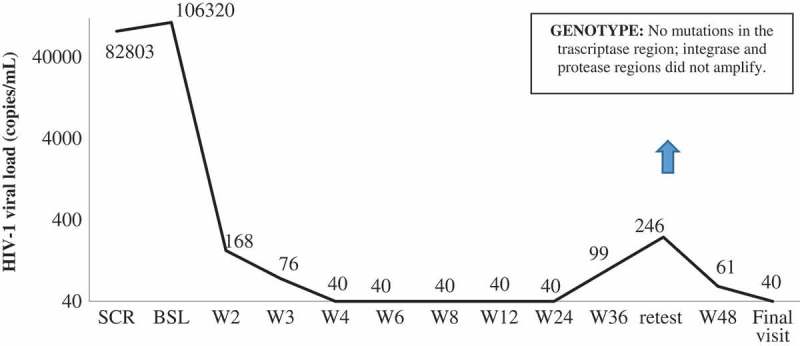
Viral load evolution (copies/mL) at virological failure by visit.

Through week 48, only one patient met the criteria for protocol-defined virologic failure. This patient entered the study with a pVL of 106,320 copies, achieved pVL below 400 copies/mL at day 10 and pVL below 50 copies/mL at week 4 and remained so until week 24. On the week 36 visit, his pVL was 99 copies/mL. After reviewing patient’s compliance with the protocol, and ruling out potential intercurrent infections or vaccinations, a retest was performed three weeks later, showing a pVL of 246 copies/mL. The patient was discontinued from the study, as mandated by the protocol, but was followed off-study until week 48. The latest pVL obtained at that time was below 50 copies/mL, without any change on his ARV regimen (Figure 3).

Albeit initially the subject denied any sex behaviour that might have put him at risk for reinfection, later on he disclosed that he had unprotected sexual intercourse with another male partner, who is HIV positive and on treatment, but still with detectable pVL. A virus pattern comparison between baseline and rebound could not be performed. Genotypic testing was performed at confirmed virological failure, but only the transcriptase region could be successfully amplified due to the low viremia. No mutations were found. Phenotypic resistance testing was not performed. The baseline genotypic test for integrase region did not reveal any mutation. A DTG plasma concentration was also obtained at failure. The DTG concentration in a sample collected 12 h after drug intake was 2.70 µg/mL, which was consistent with the pharmacokinetics and half-life reported in SPRING-1 [20] and far above the *in vitro* protein adjusted IC_90_ of viral suppression (0.064 µg/mL) [21], providing further evidence that non-adherence is not the most likely explanation for this transient viral rebound. Drug levels of 3TC were not performed.

The study drugs were well tolerated. Eight drug-related AEs were reported in six patients: headache (3), somnolence (1), epigastric pain (1), diarrhoea (1) and nausea (2) (grade 1 to 2), with no AEs leading to discontinuation of the study drug. The most frequent drug-related AE was headache in three patients. Grade 2 to 3 laboratory abnormalities (three events in three patients) were proteinuria (grade 3, since baseline), creatinine phosphokinase (grade3, only at baseline) and haematuria (grade 3, at week 48). One patient reported grade 2 insomnia at entry, without changes during the study and was deemed as not related to the study drugs.

One patient died during the study. He committed suicide, in the context of severe stress and emotional trauma. A relative disclosed that he had previous psychiatric disturbances, including a failed suicide attempt, which was not disclosed by the patient during his clinical visits. His death was noticed when he failed to show up at the week 36 visit. He achieved pVL below 50 copies/mL since week 2 and remained so until his last visit on week 24. No other SAEs were reported.

## Discussion

This pilot study was designed to test the concept that a two-drug regimen based on an INSTI and lamivudine could be considered as an option for treatment-naive, HIV-infected patients. Successful dual therapy may reduce toxicity, treatment cost and the amount of drug required to be co-formulated in single tablet treatment regimens.

Dual-therapy strategies have been studied with heterogeneous results that were summarized in two recent systematic reviews [5,22].

One of the first studies using an NRTI-sparing dual therapy was the ACTG 5142 study that compared three arms as initial ART: EFV + 2 NRTIs, ritonavir-boosted LPV/r + 2 NRTIs or EFV + LPV/r. As the dual therapy showed lower efficacy and higher risk of emergency or resistance than the others, EFV + 2 NRTIs arm became the standard of care until recent years [23]. The GARDEL study was the first one to demonstrate non-inferiority of a dual therapy compared to triple therapy at 48 and 96 weeks [24,25]. An INSTI dual regimen of DRV and raltegravir (RAL) showed non-inferiority compared to a DRV triple therapy, but failed to do so in critical populations, like patients with pVL above 100,000 copies/mL and CD4 counts below 200 cells/mL [26]. A potential explanation of these results could be the requirement of two separated doses of RAL, while DRV was dosed once daily, which could have impaired adherence. The results of the PADDLE study provide further evidence that the potency required to achieve viral suppression does not necessarily mean the use of a three-drug ARV combination. Regarding the primary endpoint, 90% of the study population had undetectable pVL at week 48. Of note, all patients, including those with baseline pVL above 100,000 copies/mL had a rapid viral suppression that matches the one seen in other studies combining DTG with two NRTIs. Compared to SINGLE and SPRING studies, VL decay did not reveal any differences between treatments [27].

The patient that presented the only SAE (suicide) achieved pVL below 50 copies/mL at week 2 and remained so until his last visit on week 24. HIV and/or HAART have been previously related to suicidality. A nationwide Swiss study showed that in 2008, suicide rates in HIV-infected individuals were more than three times higher than the rates of the general population [28]. In a voluntary testing and counselling centre in South Africa, the investigators found that a significantly elevated risk of suicidal ideation was found in 83.1% of the patients who tested seropositive [29].

EFV relationship with suicidality has been studied with divergent conclusions, ranging from a twofold increased hazard of suicidality compared with a regimen without EFV to no increased risk at all [30–32]. With DTG, events of suicidal ideation, attempt or behaviour have been reported in less than 2% of ARV treatment-naive or ARV treatment-experienced patients in eight registrational clinical trials (SPRING-1, SPRING-2, SINGLE, FLAMINGO, SAILING, VIKING, VIKING-3 and VIKING-4). These events were observed primarily in patients with a pre-existing history of depression or other psychiatric illness. Suicidal ideation is quoted in RAL and elvitegravir package inserts among the less common adverse reactions observed in treatment-experienced studies [33]. The relationship of our patient’s suicide with DTG cannot be absolutely ruled out, albeit our patient had at least two stressful and traumatic life events, as we found out retrospectively, and this could be the explanation for his tragic decision.

Through week 48, one patient met the criteria for **PDVF** (confirmed viral rebound above 50 copies after being undetectable). Therefore, the primary analysis shows 90% of the patients with pVL <50 copies in the ITT-exposed, FDA snapshot analysis. Observed failure, as the patient who committed suicide had undetectable pVL at last visit, was 5%.

The clinical implications of low-level viremia are controversial. Department of Human Health Services (DHHS) ARV guidelines define virologic failure as the inability to achieve or maintain suppression of viral replication to an HIV-RNA level <200 copies/mL and state that viremia “blips” are not usually associated with subsequent virologic failure [34].

Our study has some limitations. As a pilot proof-of-concept trial, it has a small sample size. Given that this dual regimen has been never tested before, we preferred a cautious design, treating first a cohort of 10 patients, and recruiting the second cohort when initial success was achieved, as pre-defined in the study protocol. Other limitations include being an open-label study, with a single arm and a low median VL at entry, albeit four of the volunteers had baseline pVL above 100,000 copies/mL.

A limitation for using this dual regimen is that patients with active hepatitis B infection (HBV) would not be eligible, as HBV would be exposed to 3TC monotherapy, which is no longer the standard-of-care for that infection. Universal HBV vaccination is encouraged as a public health intervention, regardless of the ARV strategy selected. Special populations like pregnant women and patients with active tuberculosis were excluded, hence this strategy cannot be considered for those populations until specific studies are completed. Nevertheless, the PADDLE study strategy might potentially address several unmet needs. First, toxicities associated with NRTIs other than 3TC may compromise lifelong exposures to those compounds. Tenofovir disoproxil fumarate (TDF) lowers bone density more than other NRTIs and can be nephrotoxic [35,36]. Abacavir can induce a potentially life-threatening hypersensitivity syndrome, and only patients who are negative for the HLA-B*5701 allele can be treated with this drug. Also, it has been associated with an increased risk of cardiovascular disease, albeit other studies failed to confirm this association [37]. The new tenofovir alafenamide formulation (TAF) has shown similar efficacy to TDF [38], with a better safety profile, but long-term data are needed to confirm the TAF safety profile. TAF is already available in western countries, but it is uncertain when it will be available in the rest of the world, which carries the heaviest burden of the epidemic. ZDV (still used in some developing countries) causes myelosuppression, gastrointestinal symptoms, headaches and mitochondrial toxicity that can lead to lipoatrophy, hepatic steatosis and lactic acidosis. Second, 3TC is a potent and inexpensive drug that can be easily co-formulated with DTG, providing a single pill QD first-line option that might be easy to administer, with minimal side effects and few drug interactions. Third, the total amount of active pharmaceutical ingredients required is low, allowing to produce smaller pills. Fourth, given the production cost of the DTG and 3TC, this regimen might be the cheapest drug combination according to a recent WHO forecast that estimated the annual cost per patient at about UDS44 [39]. Even in the US, the use of this combination as initial therapy or switching could result in significant savings. Girouard et al. compared no ART to an initial dual regimen (DTG + 3TC); to an induction–maintenance strategy (48-week induction regimen of DTG + abacavir + 3TC) followed by DTG + 3TC maintenance if virologically suppressed; and to a standard-of-care three-drug regimen (DTG + abacavir + 3TC) [40]. Two-drug regimen was the most cost-effective strategy if DTG + 3TC 48-week virologic suppression rate could exceed 90%.

## Conclusions

The results of this small, proof-of-concept, pilot study encourage further exploration of this strategy. Our results suggest that the combination of DTG and 3TC could be considered as an alternative option in first line, sparing exposure to other nucleosides and preserving other treatment options downstream, if needed. The low rate of side effects reported in the study is attributable to the avoidance of the second NRTI. Dual therapy with DTG + 3TC could be a highly effective, simple and, in some settings, cost-effective first-line option for HIV patients. If our findings are confirmed in the ongoing randomized trials GEMINI 1 (NCT02831673) and GEMINI 2 (NCT02831764), this strategy may challenge the value of adding a third nucleosid(t)ide to the outcomes of HAART. Until then, the decision of using this dual regimen outside clinical trials should be carefully considered.
